# Depression in healthcare workers with COVID-19: Prevalence and associated factors

**DOI:** 10.1192/j.eurpsy.2025.1263

**Published:** 2025-08-26

**Authors:** A. Aloui, I. Kacem, M. Bouhoula, A. Chouchane, N. Belhaj Chabbah, A. Brahem, H. Kalboussi, O. El Maalel, M. Maoua, S. Chatti

**Affiliations:** 1 Occupational Medecine Department, University Hospital of Farhat Hached, Sousse, Tunisia; 2 University of sousse, Faculty of Medecine Ibn El Jazzar, 4000 sousse, Tunisia; 3 Research laboratory LR 19SP03, Study of the risks and prospects for the prevention of non-communicable diseases in the workplace; 4 University of sousse, University Hospital of Farhat Hached; 5 Occupational medecine department, University Hospital of Sahloul, Sousse, Tunisia, Tunisia

## Abstract

**Introduction:**

Since the beginning of the health crisis caused by COVID-19, the impact of the pandemic context on the mental health of healthcare professionals has been widely studied. However, few studies have assessed psychological manifestations in healthcare workers affected by COVID-19.

**Objectives:**

Determine the prevalence and factors associated with depressive symptoms in healthcare workers with COVID-19.

**Methods:**

This is a cross-sectional analytical study of health professionals at Farhat Hached University Hospital with COVID-19 over a 6-month period from September 2020 to February 2021. Depressive symptoms were screened using the Depression Subscale of Hospital Anxiety and Depression Scale (HADS-D).

**Results:**

Our study included 477 healthcare professionals. The mean age was 39.9 ±10.8 years, with a predominance of women (sex ratio =0.27). Almost a third of the participants were nurses (32.1%), followed by doctors (27.9% of cases). Most of the healthcare workers affected developed a pauci-symptomatic form of the infection (73.8%), with an average confinement time of 14.9±6.9 days. On returning to work, 74 staff (15.5%) noticed that their colleagues were withdrawing from work. According to the HADS-D scale, depressive symptoms were observed in 19.1% of cases. The predictive factors were gender (p<10-3), age (p=0.016), the symptomatic form of COVID-19 (p= 0 ,038), the duration of confinement (p<10-3) and the withdrawn attitude experienced by colleagues on returning to work (p<10-3).

**Conclusions:**

Our results showed that psychological suffering, particularly depression, among care staff depended on several predictive factors. Psychological support during and even after the period of confinement is therefore necessary

**Disclosure of Interest:**

None Declared

